# Efficacy and Safety of Umeclidinium Added to Fluticasone Propionate/Salmeterol in Patients with COPD: Results of Two Randomized, Double-Blind Studies

**DOI:** 10.3109/15412555.2015.1034256

**Published:** 2015-10-09

**Authors:** Thomas M. Siler, Edward Kerwin, Karen Singletary, Jean Brooks, Alison Church

**Affiliations:** ^a^ Midwest Chest Consultants, PC, St. Charles, Missouri, USA; ^b^ Clinical Research Institute of Southern Oregon, Medford, Oregon, USA; ^c^ GSK, Respiratory and Immuno-Inflammation, Research Triangle Park, North Carolina, USA; ^d^ GSK, Respiratory Medicines Development Centre, Stockley Park, Uxbridge, United Kingdom

**Keywords:** bronchodilation, inhaled corticosteroid, long-acting beta agonist, long-acting muscarinic antagonist

## Abstract

Combinations of drugs with distinct and complementary mechanisms of action may offer improved efficacy in the treatment of chronic obstructive pulmonary disease (COPD). In two 12-week, double-blind, parallel-group studies, patients with COPD were randomized 1:1:1 to once-daily umeclidinium (UMEC; 62.5 μg and 125 μg)  or placebo (PBO), added to twice-daily fluticasone propionate/salmeterol (FP/SAL; 250/50 μg). In both studies, the primary efficacy measure was trough forced expiratory volume in 1 second (FEV_1_) at Day 85. Secondary endpoints were weighted-mean (WM) FEV_1_ over 0–6 hours post-dose (Day 84) and rescue albuterol use. Health-related quality of life outcomes (St. George's Respiratory Questionnaire [SGRQ] and COPD assessment test [CAT]) were also examined. Safety was assessed throughout. Both UMEC+FP/SAL doses provided statistically significant improvements in trough FEV_1_ (Day 85: 0.127–0.148 L) versus PBO+FP/SAL. Similarly, both UMEC+FP/SAL doses provided statistically-significant improvements in 0–6 hours post-dose WM FEV_1_ *versus* PBO+FP/SAL (Day 84: 0.144–0.165 L). Rescue use over Weeks 1–12 decreased with UMEC+FP/SAL in both studies *versus* PBO+FP/SAL (Study 1, 0.3 puffs/day [both doses]; Study 2, 0.5 puffs/day [UMEC 125+FP/SAL]). Decreases from baseline in CAT score were generally larger for both doses of UMEC+FP/SAL versus PBO+FP/SAL (except for Day 84 Study 2). In Study 1, no differences in SGRQ score were observed between UMEC+FP/SAL and PBO+FP/SAL; however, in Study 2, statistically significant improvements were observed with UMEC 62.5+FP/SAL (Day 28) and UMEC 125+FP/SAL (Days 28 and 84) versus PBO+FP/SAL. The incidence of on-treatment adverse events across all treatment groups was 37–41% in Study 1 and 36–38% in Study 2. Overall, these data indicate that the combination of UMEC+FP/SAL can provide additional benefits over FP/SAL alone in patients with COPD.

## Introduction

Inhaled anti-inflammatory agents and bronchodilators such as corticosteroids, muscarinic antagonists and β_2_-agonists are central to the pharmacological management of chronic obstructive pulmonary disease (COPD) (1,2). Combinations of drugs with distinct and complementary mechanisms of action may offer improved efficacy in the treatment of COPD (2), which may in turn help to relieve the burden of COPD on daily activities. Furthermore, clinical studies have shown the use of an inhaled corticosteroid/long-acting β_2_-agonist (ICS/LABA) combination together with a long-acting muscarinic antagonist (LAMA) is well tolerated in patients with COPD and associated with improvements in lung function, symptoms, and health status compared with individual ICS/LABA or LAMA therapy (3–5). These findings are reflected in the current version of Global Initiative for Chronic Obstructive Lung Disease (GOLD) guidelines, which recommend the combined use of an ICS/LABA product with a LAMA as a secondary treatment option for symptomatic patients with severe airflow obstruction and at high risk of exacerbations (2).

Fluticasone propionate plus salmeterol (FP/SAL; GSK, London, UK) is an ICS/LABA combination indicated for maintenance treatment of airflow obstruction and for reduction of exacerbations in patients with COPD (6). The LAMA umeclidinium (UMEC, GSK573719; GSK, London, UK) has recently been approved as maintenance treatment for COPD in the US and EU (7,8). Early dose-ranging studies demonstrated improvements in lung function with UMEC over a dose range of 62.5 to 1000 μg versus placebo (PBO), with no clear dose-­differentiation (9). A further dose-ranging study of UMEC with doses from 15.6 to 125 μg once daily demonstrated dose ordering, with UMEC 125 μg showing the greatest benefit in lung function and rescue use, yet similar safety profiles to the lower doses (10). To fully characterize the efficacy and safety profile of UMEC, two doses of UMEC (62.5 and 125 μg) were investigated in subsequent studies.

Here, we report the results of two studies that evaluated the efficacy and safety of once-daily UMEC (62.5 μg and 125 μg) when added to twice-daily FP/SAL (250/50 μg) in patients with COPD. The study was designed primarily to assess lung function, although other endpoints were assessed.

## Methods

### Study designs

The two studies reported here were 12-week, randomized, double-blind, parallel-group studies. Study 1 (ClinicalTrials.gov registration number: NCT01772134; GSK study number: AC4116135) was conducted in Canada, Germany, the Republic of Korea, and the US. Study 2 (ClinicalTrials.gov registration number: NCT01772147; GSK study number: AC4116136) was conducted in Chile, the Czech Republic, the Republic of Korea, Poland, and the United States.

The primary objective of both studies was to compare the efficacy and safety of UMEC (administered at a dose of 62.5 μg or 125 μg) plus FP/SAL (UMEC + FP/SAL) with PBO + FP/SAL in patients with COPD.

Eligible patients were 40 years of age or older and had an established history of COPD as defined by the American Thoracic Society/European Respiratory Society (11). Inclusion criteria were: current or former cigarette smoker with a smoking history of 10 pack-years or more; a pre- and post-salbutamol (albuterol) forced expiratory volume in 1 second (FEV_1_)-to-forced-vital-capacity (FVC) ratio < 0.70 and a post-albuterol FEV_1_ of 70% of predicted normal values or less (calculated from National Health and Nutrition Examination Survey III reference equations (12,13); and a score of 2 or higher on the modified Medical Research Council Dyspnea Scale (14) at study Visit 1. Exclusion criteria included: hospital admission for COPD or pneumonia within the 12 weeks before study Visit 1; or a present diagnosis of asthma or other known respiratory disorder. Full inclusion and exclusion criteria are described in the Supplementary materials.

Both studies were approved by the relevant local ­ethics review committees and conducted in accordance with the Declaration of Helsinki (15) and Good Clinical Practice guidelines. All patients gave written, informed consent before study participation.

### Randomization and blinding

Both studies were multicenter, randomized, double-blind, parallel-group trials. Patients entered a 4-week open-label run-in treatment with FP/SAL 250/50 μg. After the run-in period, the eligible patients were randomly assigned in a 1:1:1 ratio to once-daily UMEC 62.5 μg (delivering 55 μg), UMEC 125 μg (delivering 113 μg), or PBO administered double-blind via ELLIPTA™ dry powder inhaler, plus FP/SAL (250/50 μg administered twice daily, delivering 220/21 μg) administered open-label via DISKUS™ inhaler over a 12-week treatment period. The randomization schedule was generated using a validated computerized system (RandAll version 2.14) and patients were randomized using an Interactive Voice Response System. Concurrent use of albuterol was ­permitted as rescue medication throughout the study.

### Outcomes and assessments

For both studies, the primary efficacy measure was trough FEV_1_ at Day 85 (defined as the mean of the FEV_1_ values obtained 23 and 24 hours after dosing on Day 84). The secondary endpoints were weighted-mean (WM) FEV_1_ over 0–6 hours post-dose at Day 84 and rescue albuterol use (percentage of rescue-free days and mean number of puffs per day). The proportion of patients achieving an increase of ≥ 0.100 L above baseline in trough FEV_1_ was also assessed as a lung function ­endpoint_._

It has been reported that a change in pre-dose FEV_1_ of approximately 0.100 L can be perceived by patients (16), and therefore this was considered a clinically-meaningful improvement. Other lung function endpoints assessed were: trough FEV_1_ at Days 2, 28, 56, and 84 and WM FEV_1_ over 0–6 hours post-dose at Day 1; proportion of patients achieving an increase in FEV_1_ of ≥ 12% and ≥ 0.200 L above baseline at any time during 0–6 hours post-dose at Day 1; peak FEV_1_ on Days 1, 28, and 84; serial FEV_1_ at 15 and 30 min, 1, 3, 6, 23, and 24 hours after dosing on Days 1, 28, and 84; and serial and trough FVC.

Health-related quality of life (HRQoL) outcomes included COPD Assessment Test (CAT) and St. George's Respiratory Questionnaire (collected as SGRQ for patients with COPD, but converted to and reported as SGRQ). Baseline measures for the CAT and SGRQ were taken at Visit 2 after 4 weeks of treatment with open-label FP/SAL, consistent with the timing of other baseline assessments. A reduction in SGRQ score of 4 points was considered the minimally clinically important difference (MCID) (17). Safety and tolerability assessments included the incidence of adverse events (AEs), vital signs (including pulse rate and systolic and diastolic blood pressure), and the recording of COPD exacerbations. The incidence of AEs of special interest, including cardiovascular events, was assessed using Standardised Medical Dictionary for Regulatory Activities Queries: cardiac arrhythmias, cardiac failure, ischemic heart disease, central nervous system hemorrhages, cerebrovascular conditions, pneumonia, and lower respiratory tract infections. A COPD exacerbation was defined as an acute worsening of symptoms of COPD requiring the use of any treatment beyond study medication or rescue albuterol/salbutamol.

### Statistical analyses and sample size considerations

For both studies, sample size calculations used a two-sided significance level of 5% and an estimate of the residual standard deviation (SD) for trough FEV_1_ of 0.220 L. It was calculated that 160 patients per treatment group would provide 90% power to detect a 0.080 L difference between UMEC + FP/SAL treatment groups and PBO + FP/SAL in trough FEV_1_. To account for a 20% withdrawal rate, approximately 600 patients (200 patients per treatment) were to be randomized in each study.

The primary analysis was performed on the intent-to-treat (ITT) population, which was defined as all patients randomized to treatment who received at least one dose of study drug. The primary endpoint was analyzed using a mixed models repeated measures (MMRM) analysis (18) with baseline FEV_1_, smoking status, day (as a categorical variable) and treatment as covariates. Day-by-baseline interaction and day-by-treatment interaction were included to estimate treatment effect at each day. The model used all available trough FEV_1_ values recorded on Days 2, 28, 56, 84, and 85. The analysis of 0−6 hours’ WM FEV_1_ on Day 84 was done in a similar way. Estimated differences between each dose of UMEC + FP/SAL and PBO + FP/SAL were presented together with 95% confidence intervals and p-values. Rescue medication use (mean puffs/day over Weeks 1–12) was analyzed using an analysis of covariance model adjusting for baseline, treatment group, and smoking status.

To account for multiplicity across treatment ­comparisons and key endpoints, a step-down, closed testing procedure was used (further details are included as Supplementary Materials), as reported previously (19).

## Results

### Patients

**Figure 1.**
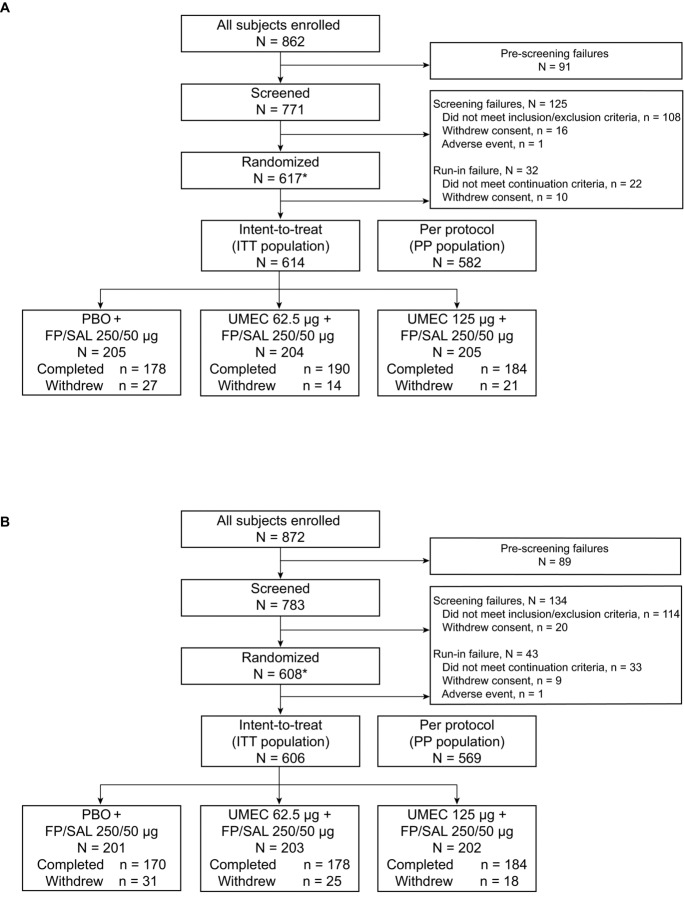
**Summary of patient disposition in Study 1 (A) and Study 2 (B).**  AE, adverse event; FP/SAL, fluticasone propionate/salmeterol combination; ITT, intent-to-treat; PBO, placebo; PP, per protocol; UMEC, umeclidinium. *The run-in population included screening failures, run-in failures, and those in the ITT population (i.e., any patient who took at least one dose of open-label FP/SAL during the run-in period). Study 1: Reasons for withdrawal: PBO + FP/SAL: AE (*n* = 6), withdrew consent (*n* = 3), lost to follow-up (*n* = 2), protocol deviation (*n* = 4), lack of efficacy (*n* = 11), subject reached protocol-stopping criteria (*n* = 1); UMEC 62.5 + FP/SAL: PBO + FP/SAL: AE (*n* = 5), withdrew consent (*n* = 1), protocol deviation (*n* = 3), lack of efficacy (*n* = 5); UMEC 125 + FP/SAL: AE (*n* = 10), withdrew consent (*n* = 5), protocol deviation (*n* = 3), lack of efficacy (*n* = 3). Study 2: Reasons for withdrawal: PBO + FP/SAL: AE (*n* = 13), withdrew consent (*n* = 7), lost to follow-up (*n* = 1), protocol deviation (*n* = 2), lack of efficacy (*n* = 8); UMEC 62.5 + FP/SAL: AE (*n* = 10), withdrew consent (*n* = 8), protocol deviation (*n* = 1), lack of efficacy (*n* = 6); UMEC 125 + FP/SAL: AE (*n* = 6), withdrew consent (*n* = 4), lost to follow-up (*n* = 1), protocol deviation (*n* = 1), lack of efficacy (*n* = 6).

In Study 1, 862 patients were enrolled across 16 centers in Canada, 33 centers in Germany, 8 centers in the Republic of Korea, and 13 centers in the US. In total, 617 patients were randomized to treatment, 614 received at least one dose of study medication and were included in the ITT population, and of these 552 (90%) completed the study (Figure 1A). In Study 2, 872 patients were enrolled across 8 centers in Chile, 11 centers in the Czech Republic, 8 centers in the Republic of Korea, 8 centers in Poland, and 17 centers in the United States. In total, 608 were randomly assigned to treatment, 606 received at least one dose of study medication and were included in the ITT population, and 532 (88%) completed the study (Figure 1B). Patient demographics and characteristics were well balanced between treatment groups and between studies, except the proportion of females in Study 2 was lower for UMEC 62.5 μg + FP/SAL compared with the other treatment groups (Table 1) and there were more current smokers in Study 1 (50−57%) than in Study 2 (36−39%).

**Table 1.** Patient demographics and characteristics (ITT population) at screening and baseline

	Study 1			Study 2		
PBO + FP/SAL 250/50	UMEC 62.5 + FP/SAL 250/50	UMEC 125 + FP/SAL 250/50	PBO + FP/SAL 250/50	UMEC 62.5 + FP/SAL 250/50	UMEC 125 + FP/SAL 250/50	
(*n* = 205)	(*n* = 204)	(*n* = 205)	(*n* = 201)	(*n* = 203)	(*n* = 202)	Age, years
Age, years	63.4 (8.27)	62.7 (7.84)	63.2 (8.95)	65.7 (7.92)	64.5 (8.31)	65.5 (7.89)
Male, *n* (%)	132 (64)	133 (65)	142 (69)	123 (61)	140 (69)	120 (59)
Current smoker at screening^a^, *n* (%)	116 (57)	102 (50)	115 (56)	77 (38)	73 (36)	78 (39)
Smoking pack-years	48.4 (25.86)	49.8 (24.53)	50.4 (27.88)	45.1 (25.73)	44.3 (27.76)	42.8 (21.76)
Post-albuterol % predicted FEV_1_	47.4 (13.34)	46.8 (12.35)	46.7 (13.12)	44.8 (13.32)	43.9 (11.53)	47.6 (12.77)
Post-albuterol FEV_1_/FVC	47.39 (10.818)	46.72 (9.758)	46.63 (10.972)	44.58 (11.117)	45.02 (10.622)	47.06 (11.076)
Baseline FEV_1,_ L	1.310 (0.4602)	1.312 (0.4745)	1.354 (0.5405)	1.137 (0.4507)^b^	1.155 (0.4419)	1.207 (0.4789)
Baseline FVC, L	2.758 (0.8382)	2.785 (0.8120)	2.854 (0.9147)	2.508 (0.7880)^b^	2.599 (0.7970)	2.592 (0.8482)
Percent reversibility to albuterol	14.5 (13.48)	16.2 (14.37)	16.2 (14.25)	13.4 (12.54)	16.1 (13.22)^c^	16.6 (14.43)
Rescue-free days at baseline, %^d^	45.4 (41.61)	46.8 (41.45)	44.4 (41.88)	39.1 (42.20)	40.9 (41.37)	37.8 (40.63)
Number of puffs of rescue medication (albuterol)/day^d^	1.9 (2.40)	1.9 (2.27)	2.1 (2.64)	2.2 (2.37)	2.4 (2.90)	2.2 (2.41)
CAT score at baseline	18.16 (7.021)	17.79 (7.404)	18.71 (6.921)	18.08 (7.425)	18.12 (7.347)	17.02 (7.075)
SGRQ score at baseline^e^	43.97 (17.558)	43.38 (17.572)	45.39 (16.145)	47.80 (17.678)	46.40 (16.713)	44.94 (15.689)
GOLD stage, n (%)						
II	100 (49)	89 (44)	91 (44)	78 (39)	71 (35)	95 (47)
III	84 (41)	100 (49)	85 (41)	92 (46)	108 (53)	88 (44)
IV	21 (10)	15 (7)	29 (14)	31 (15)	24 (12)	19 (9)
Reversible^f^ to albuterol, *n* (%)	69 (34)	80 (39)	77 (38)	55 (27)	62 (31)^c^	66 (33)
Reversible^f^ to albuterol and ipratropium^g^, n (%)	106 (52)	118 (58)	121 (59)	104 (53)	103 (52)	115 (58)
Any pre-treatment concomitant medication, n (%)	196 (96)	187 (92)	186 (91)	189 (94)	196 (97)	195 (97)
Long-acting β_2_-agonist, n (%)	114 (56)	118 (58)	113 (55)	128 (64)	132 (65)	132 (65)
Inhaled corticosteroids, n (%)	98 (48)	112 (55)	106 (52)	121 (60)	119 (59)	122 (60)
Long-acting muscarinic antagonist, n (%)	92 (45)	82 (40)	94 (46)	60 (30)	65 (32)	57 (28)

CAT, COPD assessment test; COPD, chronic obstructive pulmonary disease; FEV_1_, forced expiratory volume in 1 second; FP/SAL, fluticasone propionate/salmeterol combination; FVC, forced vital capacity; GOLD, Global initiative for Obstructive Lung Disease; ITT, intent-to-treat; PBO, placebo; SD, standard deviation; SGRQ, St. George's Respiratory Questionnaire; UMEC, umeclidinium.

Values are reported as mean (SD) unless otherwise stated.

aReclassified: Patient reclassified as current smoker if smoked within 6 months; ^b^ *n* = 200; ^c^ *n* = 202; ^d^Study 1: PBO + FP/SAL 250/50, *n* = 203; UMEC 62.5 + FP/SAL 250/50, *n* = 202; UMEC 125 + FP/SAL 250/50, *n* = 200; Study 2: PBO + FP/SAL 250/50, *n* = 196, UMEC 62.5 + FP/SAL 250/50, *n* = 202; UMEC 125 + FP/SAL 250/50, *n* = 195; ^e^Study 1: PBO + FP/SAL 250/50, *n* = 203; UMEC 62.5 + FP/SAL 250/50, *n* = 200; UMEC 125 + FP/SAL 250/50, *n* = 204; Study 2: PBO + FP/SAL 250/50, *n* = 201, UMEC 62.5 + FP/SAL 250/50, *n* = 202; UMEC 125 + FP/SAL 250/50, *n* = 198; ^f^reversible to albuterol (and to albuterol and ipratropium) was defined as an increase in FEV_1_ of ≥ 12% and ≥ 200 mL following administration of the drug(s); ^g^in Study 1,  *n* = 203 for PBO + FP/SAL 250/50, *n* = 204 for UMEC 62.5 + FP/SAL 250/50, *n* = 204 for UMEC 125 + FP/SAL 250/50; in Study 2, *n* = 198 for PBO + FP/SAL 250/50, *n* = 199 for UMEC 62.5 + FP/SAL 250/50, *n* = 200 for UMEC 125 + FP/SAL 250/50.

### Outcomes

#### Lung function

**Figure 2.**
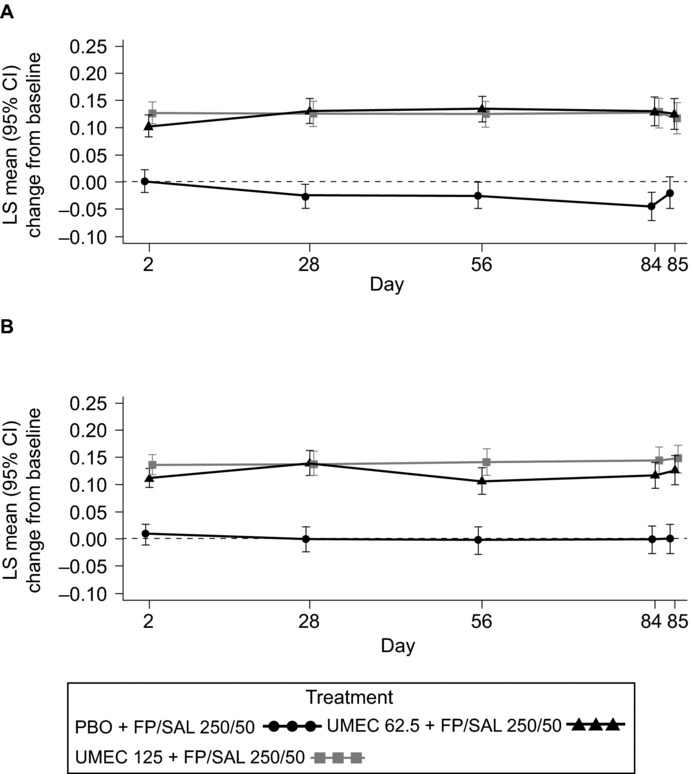
**LS mean (95% CI) change from baseline in trough FEV_1_ (L) in Study 1 (A) and Study 2 (B) (ITT).** CI, confidence interval; FEV_1_, forced expiratory volume in 1 second; FP/SAL, fluticasone propionate/salmeterol combination; ITT, intent-to-treat; LS, least squares; PBO, placebo; UMEC, umeclidinium. Analysis performed using a repeated measures model with covariates of treatment, baseline (mean of the two assessments made 30 minutes and 5 minutes pre-dose on Day 1), smoking status, Day, Day by baseline and Day by treatment interactions.

In both studies, treatment with either dose of UMEC (62.5 μg or 125 μg) + FP/SAL resulted in statistically significant and clinically-meaningful mean improvements of 0.127–0.148 L in trough FEV_1_ at Day 85 compared with PBO + FP/SAL (both *p* < 0.001; Table 2, Figure 2). Statistically significant improvements exceeding 0.100 L compared with PBO + FP/SAL were also demonstrated at Days 2, 28, 56, and 84 (either UMEC dose in either study; all *p* < 0.001; Figure 2).

**Table 2.** Lung function, rescue use and HRQoL endpoints for both studies (ITT population)

	Study 1			Study 2		
	PBO + FP/SAL 250/50 (n = 205)	UMEC 62.5 + FP/SAL 250/50(n = 204)	UMEC 125 + FP/SAL 250/50(n = 205)	PBO + FP/SAL 250/50(n = 201)	UMEC 62.5 + FP/SAL 250/50(n = 203)	UMEC 125 + FP/SAL 250/50(n = 202)
Trough FEV1 at Day 85, La	n = 177	n = 190	n = 184	n = 169	n = 178	n = 183
LS mean Difference vs PBO + FP/SAL 250/50 (95% CI)	—	0.147*(0.107, 0.187)	0.138*(0.097, 0.178)	—	0.127*(0.089, 0.164)	0.148*(0.111, 0.185)
0–6 h post-dose WM FEV1 at Day 84, L	n = 176	n = 186	n = 183	n = 171	n = 178	n = 184
LS mean difference vs PBO + FP/SAL 250/50 (95% CI)	—	0.164*(0.126, 0.203)	0.160*(0.122, 0.199)	—	0.144*(0.107, 0.182)	0.165*(0.128, 0.203)
Proportion of patients with trough FEV1 ≥ 0.100 L at Day 85	n = 204	n = 204	n = 203	n = 200	n = 203	n = 202
Odds ratio vs PBO + FP/SAL 250/50 (95% CI)	—	5.6*(3.5, 8.9)	4.5*(2.8, 7.2)	—	4.1*(2.6, 6.5)	5.7*(3.6, 9.1)
Proportion of patients with FEV1 increase ≥ 12% and ≥ 0.200 L at Day 1	n = 205	n = 204	n = 205	n = 200	n = 203	n = 202
Odds ratio vs PBO + FP/SAL 250/50 (95% CI)	—	5.5*(3.5, 8.6)	5.2*(3.4, 8.2)	—	5.3*(3.4, 8.4)	5.6*(3.6, 8.9)
Peak FEV1 at Day 84, L	n = 179	n = 190	n = 185	n = 171	n = 178	n = 184
LS mean difference vs PBO + FP/SAL 250/50 (95% CI)	—	0.186*(0.145, 0.226)	0.168*(0.127, 0.208)	—	0.147*(0.107, 0.186)	0.167*(0.128, 0.206)
Trough FVC at Day 85, L	n = 177	n = 190	n = 184	n = 169	n = 178	n = 183
LS mean difference vs PBO + FP/SAL 250/50 (95% CI)	—	0.243*(0.178, 0.308)	0.244*(0.179, 0.309)	—	0.162*(0.095, 0.230)	0.165*(0.098, 0.232)
Rescue albuterol use, Weeks 1–12						
Percentage of rescue-free days, mean (SD)	n = 184	n = 192	n = 185	n = 177	n = 192	n = 193
	49.7 (38.99)	59.4 (40.85)	56.1 (41.24)	40.7 (41.57)	49.9 (41.62)	53.8 (40.43)
Change from baseline in percentage of rescue-free days, mean (SD)	n = 184	n = 191	n = 183	n = 174	n = 191	n = 187
	4.9 (25.66)	13.3 (28.66)	11.1 (25.70)	1.9 (27.38)	8.4 (30.23)	15.2 (28.25)
LS mean difference vs PBO + FP/SAL 250/50 (95% CI), mean puffs/day	n = 184	n = 191	n = 183	n = 174	n = 191	n = 187
	—	-0.3†(-0.5, -0.1)	-0.3†(-0.5, -0.1)	—	-0.2(-0.5, 0.0)	-0.5*(-0.8, -0.3)
CAT score, Day 84						
Mean change from baseline (SD)	n = 179	n = 190	n = 185	n = 172	n = 180	n = 184
	-0.77 (5.697)	-0.81 (5.543)	-0.92 (5.112)	0.41 (5.445)	-1.31 (7.182)	-1.42 (5.880)
SGRQ score, Day 84	n = 177	n = 186	n = 183	n = 172	n = 176	n = 177
LS mean change from baseline (SE)	-2.26 (0.710)	-3.57 (0.696)	-2.77 (0.697)	-1.50 (0.778)	-3.50 (0.769)	-4.54 (0.768)
LS mean difference vs PBO + FP/SAL 250/50 (95% CI)	—	-1.32(-3.27, 0.64)	-0.51(-2.47, 1.44)	—	-1.99(-4.14, 0.16)	-3.04†(-5.19, -0.89)

CAT, COPD assessment test; CI, confidence interval; COPD, chronic obstructive pulmonary disease; FP/SAL, fluticasone propionate/salmeterol combination; FEV_1_, forced expiratory volume in 1 second; FVC, forced vital capacity; HRQoL, health-related quality of life; ITT, intent-to-treat; LS, least squares; PBO, placebo; SD, standard deviation; SE, standard error; SGRQ, St. George's Respiratory Questionnaire; UMEC, umeclidinium; WM, weighted mean.

*p < 0.001; †*p* < 0.050.

aAnalyses of trough FEV_1_ at Day 85 (primary endpoint) performed using a repeated measures model with covariates of treatment, baseline (mean of the two assessments made 30 minutes’ and 5 minutes’ pre-dose on Day 1), smoking status, Day, Day by baseline, and Day by treatment interactions.

**Figure 3.**
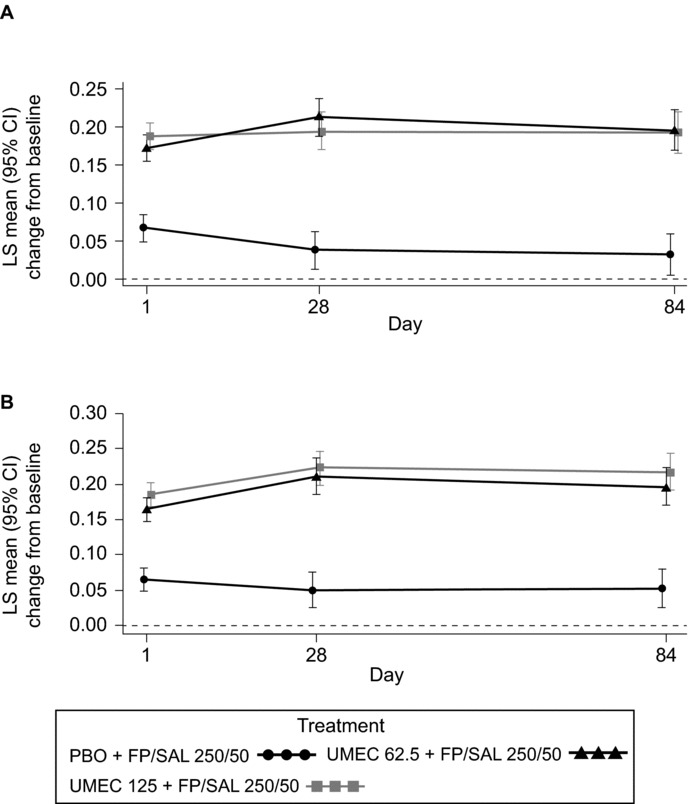
**LS mean (95% CI) change from baseline in 0–6 hours WM FEV_1_ (L) in Study 1 (A) and Study 2 (B) (ITT).** CI, confidence interval; FEV_1_, forced expiratory volume in 1 second; FP/SAL, fluticasone propionate/salmeterol combination; ITT, intent-to-treat; LS, least squares; PBO, placebo; UMEC, umeclidinium; WM, weighted mean. Analysis performed using a repeated measures model with covariates of treatment, baseline (mean of the two assessments made 30 minutes and 5 minutes pre-dose on Day 1), smoking status, Day, Day by baseline and Day by treatment interactions.

For 0–6 hours post-dose WM FEV_1_ at Day 84, treatment with either dose of UMEC (62.5 μg or 125 μg) +  FP/SAL resulted in statistically significant mean improvements of 0.144–0.165 L compared with PBO + FP/SAL in both studies (all *p* < 0.001; Table 2; Figure 3). Statistically significant improvements compared with PBO + FP/SAL were also demonstrated at Days 1 and 28 (either UMEC dose in either study; all *p* < 0.001; Figure 3).

**Figure 4.**
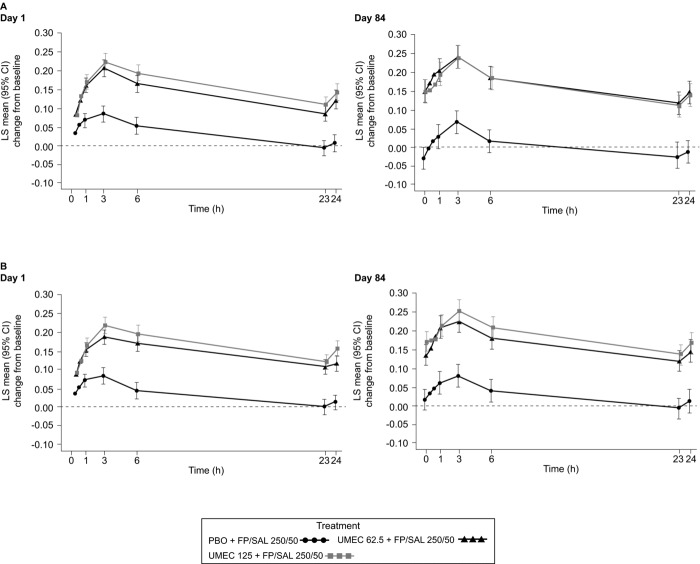
**Serial (24 hours) FEV_1_ LS mean (95% CI) change from baseline on Day 1 and Day 84 (ITT population) in Study 1 (A) and Study 2 (B).** CI, confidence interval; FEV_1_, forced expiratory volume in 1 second; FP/SAL, fluticasone propionate/salmeterol combination; ITT, intent-to-treat; LS, least squares; PBO, placebo; UMEC, umeclidinium. Analyses performed using a separate repeated measures model for each Day with covariates of treatment, baseline (mean of the two assessments made 30 minutes and 5 minutes pre-dose on Day 1), smoking status, time, time by baseline and time by treatment interactions.

Additional lung function endpoint data for both studies are presented in Table 2 and Figure 4. Compared with PBO + FP/SAL, both doses of UMEC + FP/SAL (62.5 μg or 125 μg) in both studies were associated with greater odds of having an increase in trough FEV_1_ of ≥ 0.100 L above baseline at each visit (versus not having this increase; all *p* < 0.001), greater odds of having an increase in FEV_1_ of ≥ 12% and ≥ 0.200 L  above baseline at any time during 0–6 hours post-dose at Day 1 (versus not having this increase; all  *p* < 0.001), greater improvements from baseline in peak FEV_1_ at each visit (all *p* < 0.001), greater improvements from baseline in trough FVC at each visit (all *p* < 0.001), and consistently greater serial FEV_1_ ­measurements (Figure 4). Serial FEV_1_ profiles on Day 1 and Day 84 showed sustained benefits for 24 hours post-dose. Overall, the magnitude of improvements in lung function endpoints compared with PBO + FP/SAL was similar for both doses of UMEC (62.5 μg or 125 μg) + FP/SAL.

#### Rescue use

In both studies, patients receiving UMEC + FP/SAL (62.5 μg or 125 μg) experienced a greater overall proportion of rescue-free days, and a greater increase from baseline in the proportion of rescue-free days, compared with PBO + FP/SAL (Table 2).

Statistically significant reductions in rescue use were observed for both UMEC + FP/SAL groups compared with PBO + FP/SAL in Study 1 (0.3 puffs/day, both *p* < 0.05; Table 2), but only for UMEC 125 μg + FP/SAL in Study 2 (0.5 puffs/day; Table 2).

#### HRQoL

In both studies, numerical decreases in CAT scores (denoting an improvement) were observed in all treatment groups at Day 28 and Day 84 compared with baseline, except for the PBO + FP/SAL group at Day 84 in Study 2 (Table 2). Decreases in CAT score were > 0.50 greater for all UMEC + FP/SAL groups compared with PBO + FP/SAL on both days, with the exception of Study 1 at Day 84 (Table 2).

Similarly, decreases in SGRQ total score (denoting improvement in HRQoL) were observed for all treatment groups at Day 28 and Day 84 compared with baseline (Table 2). In Study 2, the decrease from baseline in both UMEC + FP/SAL groups was close to the MCID for SGRQ (4 units). Compared with PBO + FP/SAL, in Study 1 no statistically significant differences in SGRQ score were observed with UMEC + FP/SAL (62.5 μg or 125 μg) at Days 28 or 84 (Table 2). However, in Study 2 statistically significant improvements in SGRQ score were observed for UMEC 62.5 μg + FP/SAL (Day 28 only, not Day 84) and UMEC 125 μg + FP/SAL (Days 28 and 84) compared with PBO + FP/SAL (Table 2).

#### Safety and tolerability

In Study 1, the incidence of AEs and serious AEs (SAEs) was similar across treatment groups (Table 3). In Study 2, the overall incidence of on-treatment AEs was similar across the treatment groups, but the incidence of SAEs was higher in the PBO + FP/SAL group (both UMEC + FP/SAL groups: 3%; PBO + FP/SAL: 7%). In both studies, headache and nasopharyngitis were the most commonly reported AEs across all treatment groups (3–7% and 2–5%, respectively; Table 3). Additional details on drug-related AEs and AEs of special interest are presented in the Supplementary Materials and Supplementary Table 1.

**Table 3.** Summary of on-treatment AEs reported by ≥ 3% of patients in any treatment group

	Study 1			Study 2		
	PBO + FP/SAL 250/50	UMEC 62.5 + FP/SAL 250/50	UMEC 125 + FP/SAL 250/50	PBO + FP/SAL 250/50	UMEC 62.5 + FP/SAL 250/50	UMEC 125 + FP/SAL 250/50
	(*n* = 205)	(*n* = 204)	(*n* = 205)	(*n* = 201)	(*n* = 203)	(*n* = 202)
Any on-treatment AEs, n (%)	85 (41)	78 (38)	76 (37)	74 (37)	78 (38)	73 (36)
Most common on-treatment AEs, n (%)^a^						
Headache	10 (5)	9 (4)	14 (7)	9 (4)	9 (4)	6 (3)
Nasopharyngitis	10 (5)	5 (2)	5 (2)	9 (4)	6 (3)	10 (5)
Cough	3 (1)	7 (3)	5 (2)	1 (< 1)	3 (1)	1 (< 1)
Upper respiratory tract infection	6 (3)	1 (< 1)	3 (1)	4 (2)	6 (3)	5 (2)
Back pain	5 (2)	2 (< 1)	4 (2)	5 (2)	5 (2)	6 (3)
Pneumonia	0	1 (< 1)	2 (< 1)	6 (3)	3 (1)	5 (2)
COPD	3 (1)	2 (< 1)	1 (< 1)	8 (4)	3 (1)	1 (< 1)
Any on-treatment SAEs, n (%)	8 (4)	4 (2)	6 (3)	15 (7)	6 (3)	6 (3)
Any AEs leading to permanent discontinuation of medication/withdrawal, n (%)	6 (3)	4 (2)	10 (5)	12 (6)	9 (4)	6 (3)
Fatal AEs, n (%)	0	0	1 (< 1)	1 (< 1)	1 (< 1)	0
Number of patients with a COPD exacerbation, n (%)^b^	13 (6)	9 (4)	7 (3)	20 (10)	10 (5)	8 (4)
Number of COPD exacerbations						
0	192 (94)	195 (96)	198 (97)	181 (90)	193 (95)	194 (96)
1	13 (6)	9 (4)	7 (3)	20 (10)	10 (5)	8 (4)
≥ 2	0	0	0	0	0	0

AE, adverse event; COPD, chronic obstructive pulmonary disease; FP/SAL, fluticasone propionate/salmeterol combination; PBO, placebo; SAE, serious adverse event; UMEC, umeclidinium.

aReported by ≥ 3% of patients on any treatment in either study; ^b^percentages calculated using n as the denominator.

In Study 1, one death possibly related to study drug in the UMEC 125 μg + FP/SAL group was reported in a patient with extensive cardiovascular disease (additional details provided in Supplementary Materials). In Study 2, two deaths were reported during the treatment phase; one in the PBO + FP/SAL group and one in the UMEC 62.5 μg + FP/SAL group (additional details provided in Supplementary Materials). Neither of the deaths were considered to be related to the study drug.

No clinically relevant treatment-related changes in vital signs were reported in either study. In both studies, fewer on-treatment COPD exacerbations were reported with either dose of UMEC + FP/SAL compared with PBO + FP/SAL (Table 3).

## Discussion

The findings from these two randomized, double-blind, parallel-group studies show that the addition of once-daily UMEC (62.5 or 125 μg) to twice-daily FP/SAL (250/50 μg) resulted in statistically significant and clinically-meaningful improvements in measures of lung function when compared with PBO + FP/SAL over 12 weeks in patients with COPD. A statistically significant reduction in rescue medication use, an objective measure of improvement noticeable by patients, was also observed with UMEC 125 μg + FP/SAL compared with PBO + FP/SAL in both studies, and with UMEC 62.5 μg + FP/SAL in Study 1. These findings are consistent with the results of other clinical studies of UMEC 62.5 and 125 μg, which reported improvements in trough FEV_1_, dyspnea (as measured by transitional dsypnea index), HRQoL (as measured by the SGRQ) and rescue medication use (i.e., a reduction) compared with PBO (10,20,21). This indicates that the benefit of UMEC monotherapy over PBO is maintained when administered on a background of ICS/LABA therapy.

The combined use of ICS and/or LABA or LAMA therapy is recommended as a first-line therapy for patients with symptomatic COPD and a high risk of exacerbations (2). The addition of a LAMA to ICS/LABA therapy may also provide increased efficacy compared with ICS/LABA therapy alone. This is supported by clinical study data showing that the addition of tiotropium (LAMA) to ICS/LABA therapy (budesonide/formoterol or FP/SAL) resulted in improvements in lung function, COPD symptoms, health status and severe exacerbations (i.e., a reduction) versus tiotropium alone (4,5,22–24). These studies also showed that the number and type of AEs reported with triple therapy were generally similar to those reported with dual or monotherapy agents for periods of up to one year, and were mostly related to their pharmacological mode of action.

A recent retrospective study conducted in a UK-based COPD cohort (National Health Service Tayside Respiratory Disease Information System) also assessed the impact of adding tiotropium to ICS/LABA therapy (25). This study revealed that triple therapy may reduce all-cause mortality, hospital admissions and oral corticosteroid bursts compared with ICS/LABA therapy alone. Overall, the results of this study support the rationale for LAMA/ICS/LABA triple therapy in COPD and extend the results of similar studies (3,4,26), with UMEC +  FP/SAL providing more effective bronchodilation at the beginning and end of the dosing interval compared with PBO + FP/SAL.

All three treatments (PBO + FP/SAL, UMEC 125 μg +  FP/SAL, and UMEC 62.5 μg + FP/SAL) demonstrated improvements from baseline in CAT scores. Recently, an MCID of 2 points was identified by Kon et al. (27). Although the differences from baseline reported in this study did not reach that magnitude (improvements of 0.81–1.42 for active treatments), it should be considered that the MCID was identified for a change from (untreated) baseline, whereas in these studies the baseline measurement was taken after a 4-week period on active treatment for COPD (FP/SAL). These additional improvements over FP/SAL therapy therefore represent a potentially clinically meaningful difference to PBO alone. Improvements from baseline were also noted in SGRQ scores with the three treatment groups.

However, no consistent statistically significant differences in SGRQ total score were observed between the treatment groups, with the exception of UMEC 125 μg +  FP/SAL compared with PBO + FP/SAL in Study 2. It is unclear why differences were not observed between the UMEC + FP/SAL and PBO + FP/SAL treatment groups across these two studies, as improvements in rescue use were observed. One potential reason may be that baseline SGRQ and CAT scores were measured after 4 weeks of treatment with FP/SAL. In addition, the two studies were designed to evaluate lung function efficacy ­measures, and larger studies may be required to determine a benefit on patient-reported outcomes.

Both doses of UMEC + FP/SAL were well tolerated, with the overall incidence of on-treatment AEs similar to PBO + FP/SAL in both studies. In the two studies reported here, the combination of UMEC (a LAMA) with FP/SAL (an ICS/LABA) did not result in increased cardiovascular AEs. Overall, both doses of UMEC + FP/SAL demonstrated similar efficacy and safety in the overall study population, and no substantial clinical benefit was observed with the UMEC 125-μg dose over the UMEC 62.5-μg dose when added to FP/SAL.

Although these studies demonstrate that the addition of UMEC to FP/SAL results in clinically significant improvements in lung function, they were not designed to assess the effect of the addition of UMEC to FP/SAL on exacerbations, which may require studies of longer duration and larger sample size. These studies would also provide longer-term safety data on the triple regimen. As such, the role of triple LAMA/LABA/ICS therapy compared with dual LAMA/LABA therapy in the COPD treatment paradigm is still under investigation.

## Conclusions

The findings from both studies demonstrate that the addition of UMEC 62.5 μg or

UMEC 125 μg to FP/SAL resulted in statistically significant improvements in measures of lung function compared with PBO + FP/SAL over 12 weeks. Both doses of UMEC + FP/SAL were well tolerated, with no notable treatment-related differences in AEs or changes in vital signs, and no additional safety concerns identified with the addition of UMEC to FP/SAL over a 12-week treatment period. Overall, these data suggest that patients with COPD can obtain additional benefits from the addition of UMEC to FP/SAL.

## Acknowledgments

These studies were funded by GSK. Editorial assistance was proved by Afia Akram and Joanne Ashworth of Fishawack Indicia Ltd, funded by GSK.

## Declaration of Interest Statement

EK has served on advisory boards, speaker panels or received travel reimbursement for Amphastar, AstraZeneca, Forest, Ironwood, Merck, Mylan, Novartis, Pearl, Pfizer, sanofi aventis, Sunovion, Targacept, Teva, and Theravance. He has conducted multicenter clinical research trials for approximately 70 pharmaceutical companies. TMS is part of a speaker bureau for AstraZeneca, Boehringer Ingelheim, UCB, and Novartis, and has received research support from Boehringer Ingelheim, Daiichi-Sankyo, Elevation, Forest Research ­Institute, GSK, Novartis, Pearl Therapeutics, and Sunovion. TMS also has served as a consultant for AstraZeneca and Vapotherm. AC, KS, and JB are employees of GSK and own stocks/shares in GSK.

## Supplementary Material

Supplementary_materials_1034256.doc
