# Supplementary material for: Efficacy and Safety of Umeclidinium Added to Fluticasone Propionate/Salmeterol in Patients with COPD: Results of Two Randomized, Double-Blind Studies
Source: COPD. 2015 Oct 9;13(1):1–10. doi: 10.3109/15412555.2015.1034256 (PMC4778542; doi:10.3109/15412555.2015.1034256)
Supplement: Supplementary_materials_1034256.doc [file icop_a_1034256_sm0684.doc]

**SUPPLEMENTARY MATERIALS**

**METHODS**

*Inclusion criteria*

Inclusion criteria were: an established history of COPD; ≥ 40 years of age; current or former cigarette smoker with a smoking history of 10 pack-years or more; a pre- and post-salbutamol FEV_1_-to-FVC ratio < 0.70 and a post-salbutamol FEV_1_ of 70% of predicted normal values or less (calculated from National Health and Nutrition Examination Survey III reference equations (1,2); and a score of 2 or higher on the modified Medical Research Council Dyspnea Scale (3) at study Visit 1.

*Exclusion criteria*

Exclusion criteria included: pregnancy; hospital admission for COPD or pneumonia within the 12 weeks before study Visit 1; a present diagnosis of asthma or other known respiratory disorder; hypersensitivity to any anticholinergic/muscarinic receptor antagonist, beta2-agonist, sympathomimetic, corticosteroid (intranasal, inhaled, systemic), lactose/milk protein or magnesium stearate; any medical condition that contraindicated the use of an inhaled anticholinergic; any disease that, in the opinion of the investigator, put the safety of the patient at risk through participation or that would affect the efficacy or safety analysis if the disease/condition exacerbated during the study; a history of drug or alcohol abuse within 2 years prior to Visit 1; abnormal and significant electrocardiogram findings during Visit 1.

*Statistical testing hierarchy*

For the testing hierarchy, inference for a test in the predefined hierarchy is dependent on the statistical significance (at the 5% level) of the result of the previous tests in the hierarchy, i.e., if a test in the hierarchy had a non-significant result, the results of subsequent tests were not considered strictly inferential and were presented only for descriptive purposes (e.g., *p* values were regarded as nominal). The order of the tests in the hierarchy was:

- Trough FEV_1_ at Day 85: UMEC 125 µg + FP/SAL vs PBO + FP/SAL
- 0−6 hours WM FEV_1_ at Day 84: UMEC 125 µg + FP/SAL vs PBO + FP/SAL
- Trough FEV_1_ at Day 85: UMEC 62.5 µg + FP/SAL vs PBO + FP/SAL
- 0−6 hours WM FEV_1_ at Day 84: UMEC 62.5 µg + FP/SAL vs PBO + FP/SAL

All tests in the hierarchy in both studies achieved statistical significance and inference is drawn from all other statistical tests performed.

**RESULTS**

*Safety and tolerability*

Drug-related AEs were similar for all treatment groups in Study 1 (8–11%), but lower for the PBO + FP/SAL group in Study 2 (UMEC 62.5 μg +FP/SAL, 8%; UMEC 125 μg +FP/SAL, 6%; PBO +FP/SAL: 3%; Supplementary Table 1). The incidence of AEs of special interest including cardiovascular events (cardiac failure, cardiac ischemia, stroke, and cardiac arrhythmias), pneumonia, and lower respiratory tract infections was also similar across treatment groups. With the exception of pneumonia in Study 2 (UMEC 62.5 µg +FP/SAL, 1%, UMEC 125 μg +FP/SAL, 2%; PBO +FP/SAL, 3%), no individual special-interest AE was reported in more than 1% of patients in any treatment group (Supplementary Table 1).

In Study 1, one death possibly related to study drug in the UMEC 125 μg +FP/SAL group was reported in a patient with extensive cardiovascular disease (reported cause: overdose of study medication, stroke, and cardio-respiratory arrest) during the treatment phase of the study. The patient was 150% compliant on run-in FP/SAL and 130% compliant on UMEC 125 μg +FP/SAL. In Study 2, two deaths were reported during the treatment phase; one in the PBO +FP/SAL group (cardiac arrest/severe exacerbation of COPD) and one in the UMEC 62.5 μg +FP/SAL group (abdominal tumor). Neither of the deaths were considered to be related to the study drug.

**Supplementary Table 1: Summary of drug-related AEs and AEs of special interest**

|  | **Study 1** | | | **Study 2** | | |
| --- | --- | --- | --- | --- | --- | --- |
|  | **PBO + FP/SAL  250/50**  **(*n* = 205)** | **UMEC 62.5 +**  **FP/SAL 250/50**  **(*n* = 204)** | **UMEC 125 +**  **FP/SAL 250/50**  **(*n* = 205)** | **PBO + FP/SAL 250/50**  **(*n* = 201)** | **UMEC 62.5 +**  **FP/SAL 250/50**  **(*n* = 203)** | **UMEC 125 +**  **FP/SAL 250/50**  **(*n* = 202)** |
| Any drug-related AEs, *n* (%) | 17 (8) | 16 (8) | 22 (11) | 6 (3) | 17 (8) | 12 (6) |
| AEs of special interest, *n* (%)^a^ |  |  |  |  |  |  |
| Cardiovascular – any event | 5 (2) | 2 (< 1) | 3 (1) | 3 (1) | 4 (2) | 0 |
| Cardiac arrhythmias | 1 (< 1) | 2 (< 1) | 1 (< 1) | 3 (1) | 1 (< 1) | 0 |
| Cardiac failure | 3 (1) | 0 | 1 (< 1) | 0 | 2 (< 1) | 0 |
| Cardiac ischemia | 1 (< 1) | 0 | 1 (< 1) | 1 (< 1) | 1 (< 1) | 0 |
| Stroke | 0 | 0 | 1 (< 1) | 0 | 0 | 0 |
| Pneumonia and LRTI – any event^b^ | 1 (< 1) | 2 (< 1) | 2 (< 1) | 9 (4) | 3 (1) | 5 (2) |
| LRTI excluding pneumonia | 1 (< 1) | 1 (< 1) | 0 | 2 (< 1) | 0 | 0 |
| Pneumonia | 0 | 1 (< 1) | 2 (< 1) | 7 (3) | 3 (1) | 5 (2) |

AE, adverse event; FP/SAL, fluticasone propionate/salmeterol combination; LRTI, lower respiratory tract infection; MedDRA, Medical Dictionary for Regulatory Activities; PBO, placebo; SMQ, Standardised MedDRA Queries; UMEC, umeclidinium.

^a^Assessed using MedDRA SMQs: cardiac arrhythmias assessed using cardiac arrhythmia SMQs, cardiac failure assessed using cardiac failure SMQs, cardiac ischemia assessed using ischemic heart disease SMQs, stroke assessed using central nervous system hemorrhages and cerebrovascular condition SMQs; ^b^assessed using selected MedDRA preferred terms to define pneumonia and LRTIs.

**REFERENCES**

1. Hankinson JL, Kawut SM, Shahar E, et al. Performance of American Thoracic Society-recommended spirometry reference values in a multiethnic sample of adults: the multi-ethnic study of atherosclerosis (MESA) lung study. Chest 2010; 137:138–45.
2. Hankinson JL, Odencrantz JR, Fedan KB. Spirometric reference values from a sample of the general U.S. population. Am J Respir Crit Care Med 1999; 159:179–87.
3. Manali ED, Lyberopoulos P, Triantafillidou C, et al. MRC chronic Dyspnea Scale: Relationships with cardiopulmonary exercise testing and 6-minute walk test in idiopathic pulmonary fibrosis patients: a prospective study. BMC Pulm Med 2010; 10:32.
